# An additional replication origin causes cell cycle specific DNA replication fork speed

**DOI:** 10.3389/fmicb.2025.1584664

**Published:** 2025-04-30

**Authors:** Ole Skovgaard

**Affiliations:** Department of Science and Environment, Roskilde University, Roskilde, Denmark

**Keywords:** cell cycle, DNA replication, replication fork speed, dNTP pool, *oriC*, DnaA, marker frequency analysis

## Abstract

Replication fork speed (RFS) in *Escherichia coli* has long been considered constant throughout the replication and cell cycles. In wild-type cells, the circular chromosome is duplicated bidirectionally from *oriC*, yielding two replication forks that converge at the ter region. Under slow-growth conditions, cells are smaller at initiation than at termination, so DNA replication consumes a larger fraction of cellular resources early in the cell cycle. To challenge this paradigm, we analyzed an *E. coli* strain with an additional ectopic copy of *oriC*—designated *oriX*—inserted midway along the left replichore. In this mutant, replication initiates simultaneously from both *oriC* and *oriX*, resulting in four active replication forks early in the cycle. Specifically, the rightward-moving fork from *oriX* and the leftward-moving fork from *oriC* converge first, while the leftward-moving fork from *oriX* is halted at the *terA* site until the arrival of the rightward-moving *oriC* fork. Consequently, the number of active replication forks varies dynamically—from zero to four, then two, then one, and finally zero—compared to the fixed zero–two–zero pattern observed in wild-type cells. RFS was calculated using marker frequency analysis of deep sequencing data. Our analysis revealed that RFS is reduced by approximately one third when four replication forks are active and increases by about one fourth when only one fork is active, resulting in a 2-fold variation in RFS during the replication cycle. Moreover, delaying replication initiation or increasing the available dNTP pool normalized these variations, indicating that nucleotide supply is the primary constraint on replication speed. These findings demonstrate that RFS is not inherently constant within a replication cycle and provide a basis for further studies into the factors that regulate replication kinetics.

## 1 Introduction

Accurate genome duplication is essential for all living cells. In *Escherichia coli* and most other bacteria, a circular chromosome is replicated bidirectionally at high speed. Two replisomes, assembled at the origin of chromosomal DNA replication (*oriC*), proceed along each replichore and terminate replication in the ter region opposite *oriC*, at a rate of approximately 1,000 base pairs per second with an error rate of 10^–10^ or lower (Kornberg and Baker, 1992). The timing of chromosomal DNA replication initiation is tightly controlled by several factors, including interactions of DnaA with other proteins and alterations in the nucleotide state of the DnaA protein (Katayama et al., 2010).

While most cellular components increase continuously and proportionally with cell mass during the cell cycle, the number of active replisomes changes in discrete steps (Helmstetter, 1967; Cooper and Helmstetter, 1968). In slow-growing *E. coli*, cells immediately after birth (during the B period) lack active replisomes. At the onset of DNA replication (the C period), two replisomes assemble and each replicates one chromosomal arm until they converge and terminate in the ter region. This is followed by the D period, after which the enlarged cell divides into two smaller daughter cells. Thus, neither the small newborn cells nor the large pre-divisional cells bear the energetic cost of DNA replication, whereas the medium-sized cells undergoing replication do. In faster-growing cells, the relationship between the discrete number of replication forks and the continuously increasing cell mass is considerably more complex (Cooper, 2012). Given that DNA replication imposes a significant metabolic cost (Ingraham et al., 1983), it is plausible to hypothesize that the replication fork speed (RFS) might vary with the ratio of cell mass to the number of active forks. Nevertheless, numerous studies employing DNA:DNA hybridization, transduction frequency measurements, and various labeling techniques have reported a constant replication speed [reviewed by Cooper (2012)].

However, the constant RFS is not immutable. It can be reduced under conditions of nucleotide limitation, such as low thymine concentrations in thymineless mutants (Pritchard and Zaritsky, 1970) or upon translation inhibition (Pato, 1975). In a repA mutant, the RFS was also found to decrease to 50%–60% of normal levels (Lane and Denhardt, 1975). Conversely, an oversupply of DnaA protein results in earlier initiation of replication—thus at a lower cell mass—and a concomitant reduction in RFS (Atlung et al., 1987; Løbner-Olesen et al., 1989; Skarstad et al., 1989). On the other hand, limitations in the amount or activity of DnaA (Boye et al., 1996; Morigen et al., 2003; Skovgaard and Løbner-Olesen, 2005) as well as mutations in the H-NS protein (Atlung and Hansen, 2002) have been shown to increase the RFS by up to 2-fold. A recent study by Boesen et al. (2024) manipulated the activity of the DnaA protein further by changing the ratio of DnaA-ATP to DnaA-ADP up or down. In line with the previous experiments that changed the supply of DnaA protein, a higher DnaA-ATP to DnaA-ADP ratio led to early initiation concomitant with slower RFS and the decreased DnaA-ATP to DnaA-ADP ratio led to late initiation and increased RFS.

In this study, we investigate the impact of introducing an additional chromosomal replication origin on RFS during different stages of the *E. coli* cell cycle. Using marker frequency analysis (MFA) of deep sequencing data (Skovgaard et al., 2011), we examined a strain harboring an ectopic origin. In this mutant, both origins initiate replication synchronously, thereby perturbing the cell cycle by generating additional replication forks early in slow-growing cultures. Our results indicate that the presence of extra active forks initially slows all replication forks until two forks converge, after which the remaining forks accelerate. Furthermore, we demonstrate that the primary limitation on RFS under these conditions is the increased demand for deoxynucleotide triphosphates (dNTPs) imposed by the additional replication forks.

## 2 Materials and methods

### 2.1 Bacterial strains and growth conditions

The bacterial strains and plasmids used in this study are listed in Supplementary Table 1. Cultures were grown in AB medium supplemented with phenylalanine (10 μg/ml), uracil (20 μg/ml), and a carbon source as specified in Supplementary Table 2.

Cultures were inoculated with carbon limited overnight cultures and pre-grown for at least four doublings in a shaking water-bath. Prior to reaching an OD_450_ of 0.2, these cultures were diluted 20-fold into fresh medium and allowed to grow further. Samples for DNA extractions was harvested once the OD_450_ again reached ∼0.2, and measurements continued until the OD_450_ exceeded 0.8. Doubling times were determined via log-linear regression using at least eight OD_450_ readings taken during the exponential phase.

### 2.2 DNA extraction, sequencing and replication profile plots

Genomic DNA was extracted from 1.5 ml of each harvested culture using the “Bacterial & Yeast Genomic DNA Purification Kit” (EURX, Gdańsk, Poland) and submitted to BGI (Shenzhen, China) for sequencing on an Illumina HiSeq™ 4000 platform, yielding over 10 million paired-end reads per sample. Sequencing reads were aligned to the *E. coli* MG1655 reference genome (accession no. U00096) using Bowtie2 (Langmead and Salzberg, 2012), and the resulting SAM files were binned into 1 kbp windows with an in-house script. To correct the replication profile plots, each data point was adjusted by calculating its deviation from the mean of the 30 preceding and 30 following data points within each sample; the average deviation across all samples was then applied as a correction factor. The corrected data were plotted on a semi-logarithmic scale with a log2-scaled ordinate.

### 2.3 Segmentation

**FIGURE 1:**
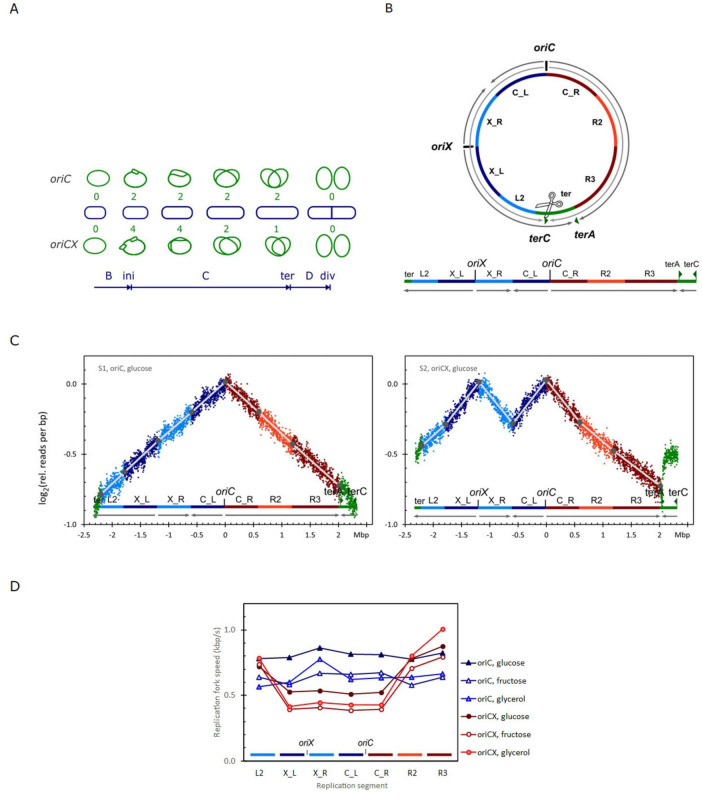
**(A)** Comparison of chromosomal DNA replication cycles in a slow growing cell (doubling time exceeds the C+D periods) initiated from either the canonical *oriC* (top) or initiated simultaneously from the canonical *oriC* and ectopic *oriX* (*oriCX*) (bottom). There are four active replication forks right after initiation in the *oriCX* strain. Two of them meet between *oriC* and *oriX* leaving two active forks. The leftward fork stalls at *terA* leaving only one active fork for the rest of the replication cycle (Dimude et al., 2018). Number of active replication forks are given with green digits and the main cell cycle events are indicated at the bottom. B, from birth to ini(tiation); C, chromosome replication; ter(mination); D, division period, from ter until div(ision). **(B)** Segmentation of the chromosome according to the number of active replication forks during the replication cycle in a slow growing *oriCX* cell. Segments named “X_” are next to *oriX*, “C_” are next to *oriC*, “L” is to the left and “R” is the right. **(C)** Replication profiles (see section “2 Materials and methods”) for *oriC* (left) and *oriCX* (right) cultivated with glucose as carbon source. Coloring relates to the segmentation in panel **(B)**. **(D)** Replication fork speed (RFS) plotted for each segment defined in panel B for *oriC* and *oriCX* cultivated with glucose [panel **(C)**], fructose or glycerol as carbon sources (replication profiles in Supplementary Figure 2).

The replication profiles were segmented based on the number of active replisomes while replicating each segment (Figure 1A) as shown in Figure 1B: Four segments are replicated simultaneously in *oriCX* with four active replisomes: X_L and X_R are replicated from *oriX* and C_L and C_R are replicated from *oriC*. These four segments are defined as having equal lengths, determined by the distance from each origin to the midpoint between the origins. The next two segments, L2 and R2, are then replicated after the segments between *oriX* and *oriC* have finished replication leaving only 2 active replisomes. The R3 segment is replicated from *oriC* with one active replisome and the ter segment is replicated in opposite directions in *oriC* and *oriCX* strains. The ter segment was extended to accommodate deviations associated with termination events.

### 2.4 Calculating RFS from marker frequency

The ratio of copy numbers N_*X*_/N_*Y*_ at fractional distances m_*x*_ and m_*y*_ from *oriC* is given by the marker frequency equation (Bremer and Churchward, 1977):


(1) $$\frac{N_{X}}{N_{Y}}=2^{\left(m_{Y}-m_{X}\right)\,C/\tau }$$

where C is the time required to replicate from the origin to the terminus, τ is the doubling time of a balanced cell culture, and m_*x*_ and m_*y*_ are fractional distances along a replichore (0 < m_*x*_ < m_*y*_ < 1 and m_*ori*_ = 0, m_*ter*_ = 1).

For m_*x*_ = m_*ori*_ and m_*y*_ = m_*ter*_ the marker frequency Equation 1, reduces to Equation 2:


(2) $$\frac{N_{X}}{N_{Y}}=2^{C/\tau }$$

The standard marker frequency equation assumes a constant RFS across the entire replichore.

However, to account for local variations, we define a local replication time C_*XY*_, for the fraction from m_*X*_ to m_*Y*_. The marker frequency for this segment is then given by Equation 3:


(3) $$\frac{N_{X}}{N_{Y}}=2^{\left(m_{Y}-m_{X}\right)\,C_{X\,Y}/\tau }$$

Taking the base-2 logarithm of both sides of Equation 3 yields Equation 4:


(4) $${\text{log}}_{2}\,\frac{N_{X}}{N_{Y}}=\left(m_{Y}-m_{X}\right)\,\frac{C_{X\,Y}}{\tau }$$

Solving Equation 4 for C_*XY*_ gives Equation 5:


(5) $$C_{X\,Y}=\tau \,\frac{{\text{log}}_{2}\,\frac{N_{X}}{N_{Y}}}{m_{Y}-m_{X}}$$

Here, C_*XY*_ represents the time required to replicate the entire replichore (of length R_*l*_) if the replication fork speed observed in the interval from m_*x*_ to m_*y*_ were maintained throughout. Thus the local RFS, *v*_*XY*_, for the fraction from m_*x*_ to m_*y*_ is given by Equation 6:


(6) $$v_{X\,Y}=\frac{R_{\ell }}{C_{X\,Y}}=\frac{R_{\ell }\,\left(m_{Y}-m_{X}\right)}{\tau \,{\text{log}}_{2}\,\frac{N_{X}}{N_{Y}}}$$

By substituting the relative positions m with absolute positions M (where M = m⋅R_*l*_), Equation 6 becomes:


(7) $$v_{X\,Y}=\frac{M_{Y}-M_{X}}{\tau \,{\text{log}}_{2}\,\frac{N_{X}}{N_{Y}}}$$

Finally, the instantaneous RFS at position M_*x*_ is defined from Equation 7 by Equation 8 as the limit when the interval shrinks to zero:


(8) $$\lim_{Y\to X}v_{X}=\frac{\text{d}\,M_{X}}{\tau \,\text{d}\,\left({\text{log}}_{2}\,N_{X}\right)}$$

This derivation provides a framework for determining the local replication fork speed from marker frequency data.

### 2.5 Determination of RFS from deep sequencing

Replication fork speed was determined from the segmented replication profiles by first defining connecting points for each segment (Figure 1C, gray dots). These connecting points were established by minimizing the root mean square deviation between the observed data points and the straight line connecting them (Figure 1C, gray lines) using an in-house script. The RFS for each segment was then calculated from the slope of its corresponding connecting line, as described by Equation 7. Finally, the overall average RFS was computed as the weighted average of the RFS values from each segment, with the ter region excluded from the analysis.

## 3 Results

Previously, we introduced an extra copy of *oriC*—designated *oriX*—approximately halfway along the left replichore of the *E. coli* chromosome. In our earlier work, we demonstrated that replication–transcription conflicts were considerably less severe in the *oriX* construct than in a similar construct where an extra *oriC* (termed *oriZ*) was inserted into the *lacZ* gene on the right replichore (Dimude et al., 2018). Both *oriX* and *oriZ* initiate bidirectional replication, which implies that, under slow-growth conditions, small cells harboring *oriX* contain four active replisomes, as opposed to the two found in wild-type cells (Figure 1A). Here, we examine the impact of this additional replication load on replication fork speed (RFS).

Wild-type *E. coli* MG1655 and strain RUC1652 carrying both *oriC* and *oriX* (hereafter referred to as the *oriC* and *oriCX* strains, respectively) were cultured to near steady state (see section “2 Materials and methods”) and analyzed via marker-frequency analysis (MFA) based on deep sequencing (Figure 1C). We have previously established that MFA is a highly sensitive method for determining RFS (Skovgaard et al., 2011), and subsequent studies have corroborated its effectiveness (Bhat et al., 2022; Huang et al., 2023).

### 3.1 RFS is reduced in cells with additional replication forks

In wild-type *E. coli* (*oriC* strain), replication profiles are nearly linear (Figure 1C, left panel). In contrast, the replication profiles of the *oriCX* strain exhibit pronounced curvature: steep slopes in the origin-proximal regions that flatten in the origin-distal regions (Figure 1C, right panel). These changes in slope correlate with the number of active replication forks, prompting us to segment the replication profiles accordingly (see section “2 Materials and methods” and Figure 1B).

The replication fork speed (RFS) for each segment was determined from the slope of its corresponding connecting line (Materials and Methods) and is displayed in Figure 1D for *oriC* cells (triangles) and *oriCX* cells (circles) grown with three different carbon sources. In the *oriC* strain, the RFS is largely constant across all segments. In the *oriCX* strain, however, the four segments near the origins replicate significantly slower than the three distal segments (Figure 1D and Supplementary Table 3). To facilitate comparison, we calculated an O-P/O-D ratio, which compares the RFS of origin-proximal (O-P) regions to that of origin-distal (O-D) regions. An O-P/O-D ratio of 1 indicates equivalent RFS in both regions, whereas a ratio below 1 indicates a slower RFS near the origin(s) relative to distal regions.

*oriC* cultures exhibited O-P/O-D ratios close to 1 (ranging from 0.97 to 1.07), with the exception of sample S15 (1.27). In contrast, the *oriCX* cultures showed O-P/O-D ratios between 0.48 and 0.54, with one outlier (sample S2, 0.66) (Supplementary Table 3). These results collectively support that, while *oriC* cells maintain a constant RFS throughout the replication cycle, the *oriCX* cells experiences nearly a 2-fold reduction in RFS during the early replication stage when all four replication forks are active, compared to later stages when only two or one fork is active.

The absence of curvature in *oriC* replication profiles, along with the alignment of profile bends with replication termination events in the *oriCX* strain, strongly suggests that the additional replication forks in the *oriCX* strain impose a limitation on RFS across all replication forks.

### 3.2 The two origins are equally efficient in the *oriCX* strain

The nearly identical read densities at *oriX* and *oriC* in *oriCX* cultures (*oriX*/*oriC* ratio 0.98–1.00; Supplementary Table 3) indicate that both origins initiate replication with equivalent efficiency. If one origin were more efficient than the other, its read density would be higher.

### 3.3 Both origins initiate in synchrony within each cell in the *oriCX* strain

Even with equal efficiency, there remains the possibility that in a subset of cells only one of the two origins might initiate replication. To address this, we simulated replication profiles for cultures in which a fraction of cells experienced initiation failure at one origin. These simulations revealed that such failures lead to a flatter slope in the replication profile for the segments between *oriX* and *oriC* (specifically, the X_R and C_L sections) compared to the segments outside the origins (X_L and C_R; see Supplementary Figure 1). A reduced slope in these regions would result in an apparently increased RFS.

To quantify initiation synchrony, we calculated a synchrony index by dividing the apparent RFS between the two origins (converging segments X_R and C_L) by the RFS of the diverging segments (X_L and C_R) (Supplementary Table 3). A synchrony index of 1 indicates that both origins initiate successfully; values above 1 suggest initiation failure at either one origin. In our experiments, the RFS values derived from the slopes of the four origin-proximal segments (X_L, X_R, C_L, and C_R; Figure 1B) were nearly identical for each sample—0.51–0.54 kbp/s for glucose-grown cultures, 0.38–0.41 kbp/s for fructose-grown cultures, and 0.42–0.44 kbp/s for glycerol-grown cultures. Additionally, the synchrony index varied from 0.99 to 1.03 (Supplementary Table 3), indicating that both origins successfully initiated replication in almost all cells.

The sharp break in the replication profile at the lowest point between *oriC* and *oriX* (Figure 1C, right panel and Supplementary Figure 2A, right panels) further supports synchronous initiation, as all replication forks converge precisely at that point. A randomly delayed initiation at one of the origins would have resulted in a flattened profile due to replication forks meeting off-center. Moreover, the replication profile of the replichore left of *oriX* extends past *terC* and is interrupted just before *terA*. This pattern suggests that termination events at *terC* are absent and that all replisomes instead converge at *terA*. The inverted slope observed between *terA* and *terC* in the *oriCX* strain compared to the *oriC* strain (Figure 1C, right panel and Supplementary Figure 2A, right panels) indicates that this region is now replicated as an extension of the left replichore originating from *oriX*.

Collectively, the symmetry of the replication profiles at each origin, the precise convergence of replication forks, and the absence of termination events at *terA* confirm our earlier conclusion that both origins initiate in synchrony in the *oriCX* strain (Dimude et al., 2018). These findings are consistent with the initiation cascade model (Løbner-Olesen et al., 1994), which predicts that all active origins within a single cell initiate within a very short time interval, resulting in initiation synchrony.

### 3.4 Reduced origin proximal RFS is not caused by pyrimidine depletion in the *oriCX* strain

**FIGURE 2:**
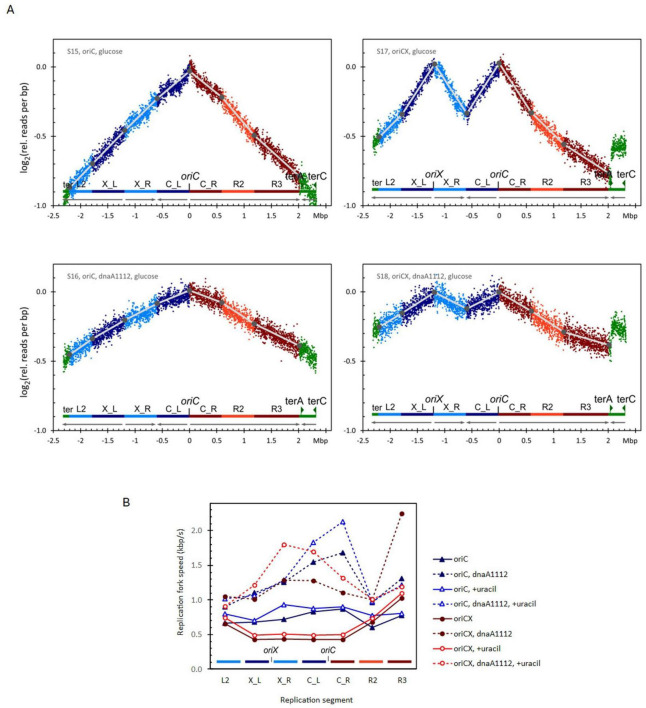
**(A)** Replication profiles for *oriC* (left), *oriCX* (right), *dnaA* (wt) (top), and *dnaA1112* (bottom) cultivated with glucose as carbon source. Additional replication profiles in Supplementary Figures 2B, C. **(B)** Replication fork speed (RFS) plotted for each segment of *oriC* and *oriCX* combined with *dnaA* (wt) or with *dnaA1112*. Strains were cultivated with glucose as carbon source and either with or without supplemented uracil. Additional RFS plot in Supplementary Figure 3.

The *E. coli* strains used in this study carry the *rph*-1 frameshift mutation, which is known to reduce pyrimidine pools available for DNA replication (Jensen, 1993). Consequently, the observed reduction in replication fork speed (RFS) when additional replisomes are active might be attributed to an increased demand for pyrimidines. To test this possibility, both *oriC* and *oriCX* strains were grown in medium supplemented with uracil (Figure 2B and Supplementary Figure 2C, upper panels).

In uracil-supplemented conditions, the average RFS increased by 12% in the *oriC* strain (0.82 kbp/s vs. 0.73 kbp/s) and by 14% in the *oriCX* strain (0.61 kbp/s vs. 0.54 kbp/s). However, the O-P/O-D ratio—a comparison of origin-proximal to origin-distal RFS—remained reduced [0.58 in the uracil-supplemented culture versus 0.54 in the unsupplemented control; Supplementary Table 4, samples with a *dnaA* (wt) allele]. This suggests that limited pyrimidine pools have, at most, a marginal effect on the RFS reduction observed in the origin-proximal segments of the *oriCX* strain.

Additionally, uracil supplementation decreased the doubling times of both strains (*oriC*: 54.6 min vs. 60.3 min; *oriCX*: 59.8 min vs. 67 min; Supplementary Table 4). Together with the increased RFS, these findings indicate that growth in medium lacking uracil is compromised by pyrimidine limitation, but this limitation does not account for the reduced RFS observed in the *oriCX* strain.

### 3.5 Postponing replication initiation with the *dnaA1112* mutation alleviate RFS variations

The reduction in replication fork speed (RFS) observed with additional active replisomes led us to hypothesize that increasing the initiation mass (Donachie, 1968) might relieve the bottleneck in the limiting resource. The *dnaA1112* mutation—originally isolated as a suppressor of the *dnaX2016* (Ts) phenotype—has been shown to increase the average cell mass (81 vs. 73 units by light scatter), reduce the number of replication origins per cell, and increase the initiation mass by 43% (Skovgaard and Løbner-Olesen, 2005). These properties indicate that replication initiation is postponed in the cell cycle in the *dnaA1112 mutant*, and once initiated, replication proceeds more rapidly. Moreover, *the dnaA1112 mutant* exhibited superior initiation synchrony measured by an asynchrony index (Boye and Løbner-Olesen, 1990) of 0.03 vs. 0.01 in wild type (Skovgaard and Løbner-Olesen, 2005). The low asynchrony index suggest that the mutant DnaA protein can also coordinate synchronous initiation of both origins in the *oriCX* strain.

To evaluate the effect of the *dnaA1112* mutation on RFS, we introduced this mutation into both *oriC* and *oriCX* strains and analyzed their replication profiles in minimal medium supplemented with either glucose (Figure 2A), glucose with uracil (Supplementary Figure 2C), or glycerol (Supplementary Figure 2B). In both strains, the *dnaA1112* mutation reduced the slopes of all replication profile segments (compare the lower panels of Figure 2A with the upper panels). Even after accounting for increases in doubling times (ranging from 2% to 18%; see Supplementary Tables 4, 6), the average RFS—calculated from these slopes—was significantly increased in both *oriC* and *oriCX* strains (compare dotted with solid line data in Figure 2B and Supplementary Figure 3).

In the *oriC* strain, the *dnaA1112* mutation increased the average RFS by 67% (from 0.73 to 1.22 kbp/s; Supplementary Table 4), consistent with our previous observation of a reduced C period [from 49 to 31 min; Skovgaard and Løbner-Olesen (2005)]. Similar increases were observed in glucose medium supplemented with uracil (55% increase) and in glycerol medium (57% increase). In glucose medium, the O-P/O-D ratio (comparing origin-proximal to origin-distal RFS) for the *dnaA1112 oriC* strain was higher (1.46 and 1.78) than for the wild-type *dnaA* allele (1.24 and 1.11; Supplementary Table 4), whereas in glycerol medium the ratio was near unity (1.01 and 1.03; Supplementary Table 6), suggesting that the *dnaA1112* mutation may provide a “kick-start” for the replication forks.

In the *oriCX* strain, the synchrony index was reduced to 0.73–0.83 in the *dnaA1112* background, compared with 0.99–1.03 for the wild-type allele (Supplementary Tables 4, 6). Based on our simulations (Supplementary Figure 1), a synchrony index around 0.78 is expected if approximately 25% of cells fail to initiate at one of the two origins, indicating that the *dnaA1112* mutation leads to initiation failure at one origin in roughly one quarter of the *oriCX* cells. Because such failures could artifactually reduce the slopes of converging segments between the origins (X_R and C_L) and thus overestimate their RFS, we calculated the average RFS and O-P/O-D ratios for *oriCX* samples using only the L2, X_L, C_R, and R2 segments (Supplementary Table 3).

Overall, the *dnaA1112* mutation increased the average RFS in the oriCX strain by 119% (from 0.53 to 1.17 kbp/s) in glucose medium, by 92% (from 0.61 to 1.16 kbp/s) in uracil-supplemented glucose medium (Supplementary Table 4), and by 61% (from 0.53 to 0.86 kbp/s) in glycerol medium (Supplementary Table 6). Furthermore, in the *dnaA1112 oriCX* strain, the RFS of origin-proximal segments increased to levels equal to or greater than those of the origin-distal segments, as reflected by an increase in the O-P/O-D ratio from 0.55–0.67 [*dnaA* (wt)] to 1.03–1.32 (*dnaA1112*; Supplementary Tables 4, 6). These results indicate that the *dnaA1112* mutation not only elevates the overall RFS—by 55%–67% in *oriC* strains and 61%–119% in *oriCX* strains—but also completely removes the bottleneck that reduces RFS in origin-proximal segments in the *oriCX* strain. In contrast to the *oriC* strain, where replication may fail if the single origin does not fire, the *oriCX* strain can complete DNA replication even if initiation fails at one origin. The reduced synchrony index suggest that initiation fails at either one of the two origins in about one quarter of the cells in *dnaA1112*, *oriCX* cells in contrast to *dnaA* (wt), *oriCX* cells where the fraction of initiation failures is below the detection limit.

### 3.6 Increasing the dNTP pool with the *nrdR*::Kan mutation alleviate RFS variations

The rate of DNA replication is dependent on the availability of deoxynucleotide triphosphates (dNTPs), which are synthesized by ribonucleotide reductase (RNR). RNR expression is regulated by several factors, including the DnaA protein, the metabolic status of the cell, and the NrdR repressor (Reyes-Lamothe and Sherratt, 2019). Deletion of *nrdR* increases transcription of the RNR regulon (Torrents et al., 2007), thereby enlarging the dNTP pool and accelerating DNA replication (Zhu et al., 2017). If the reduced RFS observed in the origin-proximal segments of the *oriCX* strain is due to limited nucleotide availability, deletion of *nrdR* should preferentially elevate RFS in these regions.

**FIGURE 3:**
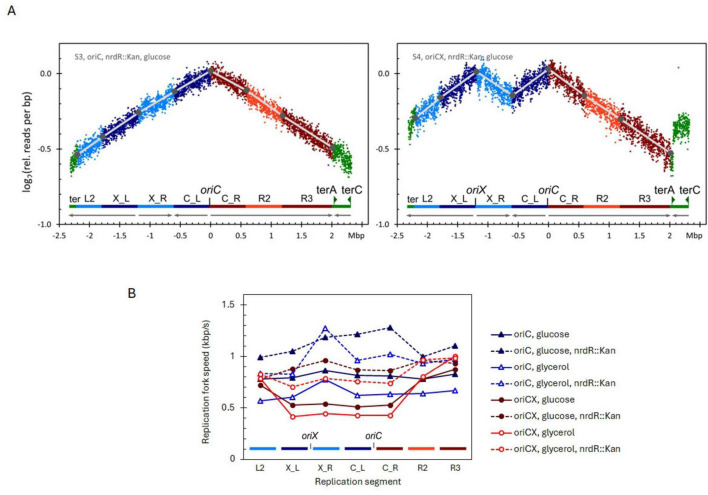
**(A)** Replication profiles for *oriC, nrdR*::Kan (left), *oriCX*, *nrdR*::Kan (right), cultivated with glucose as carbon source. Compare these profiles with the respective profiles for *nrdR* (wt) in Figure 1. Additional replication profiles in Supplementary Figure 2D. **(B)** Replication fork speed (RFS) plotted for each segment of *oriC* and *oriCX* combined with *nrdR* (wt) or *nrdR*::Kan. Strains were cultivated with either glucose or glycerol as carbon source.

To test this, we introduced the *nrdR*::Kan allele into both *oriC* and *oriCX* strains and compared their growth and replication parameters with isogenic *nrdR* (wt) strains in media containing either glucose or glycerol. In the *oriCX*, *nrdR*::Kan strain, the slopes of the replication profile segments were nearly uniform, in stark contrast to the pronounced differences seen in the *oriCX*, *nrdR* (wt) strain (compare Figure 3A with Figure 1C and Supplementary Figure 2D with the upper panel of Supplementary Figure 2B). RFS values were derived from these segmented slopes and are summarized in Supplementary Table 5.

In glucose-grown cultures, *nrdR*::Kan increased the average RFS by 38% in the *oriC* strain and by 43% in the *oriCX* strain. In glycerol-grown cultures, the average RFS increased by 50% in the *oriC* strain and by 47% in the *oriCX* strain (Supplementary Table 5). The O-P/O-D ratio also improved: in glucose medium, it increased from 1.01 to 1.17 in the *oriC* strain and from 0.70 to 1.00 in the *oriCX* strain; in glycerol medium, the ratio rose from 0.97 to 1.03 in the *oriC* strain and from 0.53 to 0.80 in the *oriCX* strain (Supplementary Table 5).

Importantly, the *nrdR*::Kan mutation did not compromise initiation synchrony in the *oriCX* strain. Instead, the synchrony index showed a slight increase—from 0.99/1.03 to 1.05/1.07 for glucose- and glycerol-grown samples, respectively (Supplementary Table 5).

In summary, the *nrdR*::Kan mutation increased the RFS across all segments in both *oriC* and *oriCX* strains. In the *oriC* strain, the enhancement was uniform across segments, whereas in the *oriCX* strain the increase was most pronounced in the origin-proximal segments, effectively equalizing their RFS with that of the origin-distal segments when grown on either glucose or glycerol. Although the magnitude of the increase was lower than that observed with the *dnaA1112* mutation, *nrdR*::Kan has the advantage of preserving initiation synchrony in the *oriCX* strain.

## 4 Discussion

A constant replication fork speed (RFS) in *E. coli* has long been considered a dogma, supported by limited experimental data from diverse methods such as autoradiographic analysis of pulse-labeled DNA fragments, gene frequency measurements, and various transduction experiments (Cooper, 2012). Although the typical RFS in bacteria is remarkably high compared to that in eukaryotic cells, several mutants have been shown to further increase RFS (Boye et al., 1996; Atlung and Hansen, 2002; Morigen et al., 2003; Skovgaard and Løbner-Olesen, 2005). However, the biological rationale for tightly controlling RFS rather than maximizing it, as well as the consequences of an increased replication fork speed, remain poorly understood.

The advent of marker frequency analysis (MFA) based on deep sequencing (Skovgaard et al., 2011) has significantly improved the resolution for measuring RFS, enabling detailed analysis over smaller segments of each replichore. In parallel, the possibility of incorporating an additional replication origin—such as *oriZ* (Wang et al., 2011)—prompted investigation into systems where replication burden varies with cell size; small cells would bear a higher replication load, while larger cells would experience a reduced burden. However, the *oriZ* construct on the right replichore encountered challenges due to replication fork collisions with highly transcribed ribosomal RNA operons (Ivanova et al., 2015). To overcome this, an ectopic origin was introduced on the left replichore (*oriX*), and both *oriC* and *oriX* were confirmed to be active (Dimude et al., 2018).

MFA of the wild-type *E. coli* MG1655 (*oriC* strain) revealed no significant RFS variations during the replication cycle, thereby supporting the concept of a constant RFS. In contrast, the *oriCX* strain—harboring both *oriC* and *oriX*—exhibited substantial variability in RFS. Specifically, RFS was nearly halved in the early replication cycle when four replication forks were active compared to later stages where only one or two forks operated. Notably, the introduction of either the *dnaA1112* or *nrdR*::Kan mutation eliminated this RFS bottleneck, whereas supplementation with uracil did not produce a similar effect.

These findings underscore that the regulation of RFS is sensitive to the number of active replication forks and can be modulated by genetic factors affecting initiation timing and nucleotide availability. Understanding these mechanisms may provide new insights into how cells balance replication efficiency with genomic stability.

### 4.1 The replication model for the *oriCX* strain

For accurate inference of RFS from replication profile slopes, the underlying model of marker frequency analysis (MFA) must be valid. In our applied model (Figure 1A) for the *oriCX* strain, it is assumed that both origins initiate synchronously in all cells, with converging replication forks—designated X_R and C_L in Figure 1B—meeting and resolving at the midpoint between *oriC* and *oriX*, while the remaining forks converge and resolve at *terA*.

We assessed both the efficiency and synchrony of initiation from the two origins in the *oriCX* strain. Our results indicate that both origins initiate synchronously and efficiently in all *oriCX* samples, with the sole exception of the *oriCX*, *dnaA1112* strain. In this strain, the synchrony index revealed initiation failure at one of the origins in approximately 25% of the cells. This observation also calls into question the exceptionally high RFS (exceeding 2 kbp/s) inferred for segment R3. A hypothetical leakage of replisomes at *terA* for those initiated from *oriX* could, for instance, lower the slope of this segment and lead to an erroneously elevated RFS estimate.

Consequently, to mitigate potential artifacts, we determined RFS variation of the origin-proximal to origin-distal (the OP-OD ratio) in the *oriCX* strain solely from the X_L, C_R, L2, and R2 segments (Figure 1).

### 4.2 Nucleotides

The *dnaA111*2 mutation was originally isolated as a suppressor of the *dnaX2016* (Ts) phenotype. Its suppressive mechanism is attributed to a reduced initiation frequency that increases the initiation mass, thereby allowing the partially defective DNA PolIII holoenzyme to complete replication. Specifically, the *dnaA1112* mutation substitutes Thr291 with Asn in the DnaA1112 protein. Thr291 is located in a β-sheet adjacent to Leu290—a residue now recognized for mediating head-to-tail interactions in DnaA complexes and essential for the release of ADP prior to rejuvenation (Sugiyama et al., 2019). The resulting impairment in rejuvenation is thought to prevent DnaA from repressing transcription of the *nrdAB* operon, thereby increasing the pool of available deoxynucleotides (Gon et al., 2006). Additionally, a higher DnaA-ADP/DnaA-ATP ratio, due to the lack of rejuvenation, may further stimulate the *nrdAB* operon operon (Augustin et al., 1994; Olliver et al., 2010), although the precise consequences of the altered head-to-tail interaction remain uncertain.

The observed ∼1.5-fold and 2-fold increases in average RFS for *oriC* and *oriCX* strains, respectively (Supplementary Table 4), likely result from a combination of factors: a decreased number of active replication forks per cell mass, diminished repression, and increased activation of the *nrdAB* operon—all of which boost the availability of deoxynucleotides for each active fork.

To distinguish the effects of an increased initiation mass from those of enhanced nucleotide availability, we increased transcription of nucleotide reductase genes by inactivating the *nrdR* gene, which encodes the NrdR repressor (Torrents et al., 2007), using a kanamycin resistance insertion (*nrdR*::Kan). Inactivation of NrdR relieved the RFS suppression observed in the origin-proximal segments of the *oriCX* strain (Figure 3B, dashed circles versus solid line circles), indicating that nucleotide availability is the primary limitation in these regions. Moreover, the average RFS increased by 38%–50% in both *oriC* and *oriCX* strains. A similar increase in RFS was reported by Zhu et al. (2017) upon induction of the *nrdAB* operon from a TetR-controlled P*_*LtetO*_* promoter.

### 4.3 Replication speed and stress

Balanced dNTP pools are crucial for maintaining the high fidelity of DNA polymerase III, as imbalances can lead to increased mutagenesis and chromosomal instability (Mathews, 2006). Gon et al. (2011) demonstrated that elevated dNTP levels directly shift the exo/pol balance of the Pol III holoenzyme, thereby increasing translesion synthesis rather than altering the activity of specialized polymerases. Consequently, while boosting dNTP pools can increase replication fork speed (RFS), this acceleration may come at the cost of reduced replication fidelity. In the *oriCX* strain, dNTP limitations early in the replication cycle—when additional replication forks are active—could similarly compromise the accuracy of DNA replication.

Moreover, the ribonucleotide reductase (RNR) encoded by *nrdAB* co-localizes with the replisome (Sánchez-Romero et al., 2010) and likely forms part of the replication complex. The observed increase in RFS in the *oriCX* strain after the convergence of replication forks suggests that RNR released from terminated forks may be transferred to still-active forks, thereby enhancing their activity.

Future studies employing deeper sequencing to yield higher-resolution MFA data in *oriCX* strains could provide valuable insights into the kinetics of RNR transfer between replication forks, further elucidating the relationship between nucleotide availability and replication dynamics.

### 4.4 Concluding remarks

In this study, bacterial growth rates were modulated by selecting different carbon sources rather than by varying carbon concentrations, temperature, or other parameters. This approach allowed us to achieve steady-state growth at sufficiently slow rates such that the doubling time exceeded the replication period (the C period; Figure 1A). Under these slow-growth conditions, terminations from previous replication rounds do not interfere with ongoing replication forks in the current cycle. In contrast, growth in richer media leads to overlapping replication cycles, which can obscure the relationship between cell size and the number of active replication forks. To confirm the robustness of our findings, we replicated key experiments across different growth rates and repeated the most critical samples, as detailed in Supplementary Table 3.

Previous studies Milbredt et al. (2016), Bruhn et al. (2018), Galli et al. (2019) applied MFA to replicons with additional origins; however, they did not specifically address RFS. For example, the data from Milbredt et al. (2016) were too noisy for detailed RFS analysis, and both Bruhn et al. (2018), Galli et al. (2019) grew cultivated *Vibrio cholerae* in rich medium, where overlapping replication cycles obscured the relationship between cell size and replication fork number. More recently, Huang et al. (2023) compared RFS in wild-type *V. cholerae* (which possesses two chromosomes) with that in the MCH1 strain (in which the two chromosomes are fused and only one origin is active) and found that RFS was faster in the MCH1 strain, where fewer replication forks are active. Similarly, Bhat et al. (2022) examined temperature-induced oscillations in RFS in *E. coli* MG1655 grown in rich medium but did not correlate these variations with the number of active replication forks per cell mass.

By applying MFA to the *oriCX* strain, we established a unique system in which RFS varies up to 2-fold across different chromosomal regions. Our data indicate that the reduced RFS in the early part of the replication cycle in the *oriCX* strain is primarily due to limited nucleotide supply when the number of active replication forks per cell mass is high. In the wild-type *oriC* strain, only two replication forks are active early in the cycle, whereas in the *oriCX* strain, four forks are active—each operating at roughly two-thirds of the speed observed in the *oriC* strain. Consequently, even though the number of active forks doubles, the overall DNA synthesis rate per cell increases by only about one-third. These findings suggest that the long-held dogma of a “constant DNA synthesis rate per cell” more accurately reflects the cellular reality than the notion of a “constant DNA synthesis rate per replication fork.” Earlier observations of a constant replication fork speed did not account for conditions in which the number of active forks is experimentally manipulated.

Our system thus provides a valuable platform for further investigations into additional factors affecting RFS and the potential consequences of RFS variations on mutation rates. Notably, when cultures are grown slowly, each chromosomal region exhibits a location-specific RFS—a phenomenon that may be masked under fast-growth conditions.

## Data Availability

The sequence data analyzed in this study is publicly available. This data can be found here: https://www.ncbi.nlm.nih.gov/, accession number: PRJEB86591.
