# Supplementary material for: An additional replication origin causes cell cycle specific DNA replication fork speed
Source: Front Microbiol. 2025 Apr 30;16:1584664. doi: 10.3389/fmicb.2025.1584664 (PMC12075136; doi:10.3389/fmicb.2025.1584664)
Supplement: Supplementary file 1 [file Data_Sheet_1.pdf]

Supplementary Material

for

An Additional Replication Origin Causes Cell Cycle Specific DNA Replication Fork Speed.

Supplementary Table S1. *E. coli* K-12 strains used

Strain	Construction: genotype	Reference
RUC1112	*lac*, *thi*, *str*, *dnaX2016*, *tna2123*::Tn10, *dnaA1112*	(Skovgaard and Løbner-Olesen, 2005)
JD20587	*rrnB3*, Δ*lacZ4787*, *hsdR514*, Δ(*araBAD*)*567*, Δ(*rhaBAD*)*568*, *rph*-1, *nrdR*::*kan*	(Baba et al., 2006)
MG1655	*rph*-1	From Martin Marinus
RUC1593	*rph*-1, Δ*lacU169 gal490* λcI_{857} Δ(*cro-bioA*) *pheA*::*oriX*-cat	(Dimude et al., 2018)
RUC1652	MG1655 x P1(RUC1593): *rph*-1, *pheA*::*oriX-cat*	This work
RUC1660	MG1655 x P1(JD20587): *rph*-1, *nrdR*::*kan*	This work
RUC1661	RUC1652 x P1(JD20587): *rph*-1, *pheA*::*oriX-cat*, *nrdR*::*kan*	This work
RUC1662	MG1655 x P1(RUC1112): *rph*-1, *tna2123*::Tn10, *dnaA1112*	This work
RUC1665	RUC1652 x P1(RUC1112): *rph*-1, *pheA*::*oriX-cat*, *tna2123*::Tn10, *dnaA1112*	This work

Supplementary Table S2. Genotypes, growth medium and doubling times of all samples

Sampl e	Strain	Origins	Mutations	C source, nucleobase[1])	τ (min)	Replication profile; RFS (Figures)	Data analysis (Table)
**S1**	MG1655	*oriC*		Glucose	56.3	1C; 1D, 3B	S3, S5
**S2**	RUC1652	*oriCX*		Glucose	62.7	1C; 1D, 3B	S3, S5
**S3**	RUC1660	*oriC*	Δ*nrdR*	Glucose	59.2	3A; 3B	S5
**S4**	RUC1661	*oriCX*	Δ*nrdR*	Glucose	65.5	3A; 3B	S5
**S5**	MG1655	*oriC*		Fructose	79.3	S2; 1D	S3
**S6**	RUC1652	*oriCX*		Fructose	75.3	S2; 1D	S3
**S7**	MG1655	*oriC*		Glycerol	87.7	S2; 1D, 3B	S3, S5
**S8**	RUC1652	*oriCX*		Glycerol	92.1	S2; 1D, 3B	S3, S5
**S9**	RUC1660	*oriC*	Δ*nrdR*	Glycerol	94.4	S2; 3B	S5
**S10**	RUC1661	*oriCX*	Δ*nrdR*	Glycerol	98.7	S2; 3B	S5
**S11**	MG1655	*oriC*		Glycerol	91.0	S2; S3	S3, S6
**S12**	RUC1662	*oriC*	*dnaA1112*	Glycerol	107.4	S2; S3	S6
**S13**	RUC1652	*oriCX*		Glycerol	95.6	S2; S3	S3, S6
**S14**	RUC1665	*oriCX*	*dnaA1112*	Glycerol	107.2	S2; S3	S6
**S15**	MG1655	*oriC*		Glucose	60.3	2A; 2B	S3, S4
**S16**	RUC1662	*oriC*	*dnaA1112*	Glucose	67.0	2A; 2B	S4
**S17**	RUC1652	*oriCX*		Glucose	64.5	2A; 2B	S3, S4
**S18**	RUC1665	*oriCX*	*dnaA1112*	Glucose	65.9	2A; 2B	S4
**S19**	MG1655	*oriC*		Glucose+Ura	54.6	S2; 2B	S4
**S20**	RUC1662	*oriC*	*dnaA1112*	Glucose+Ura	60.4	S2; 2B	S4
**S21**	RUC1652	*oriCX*		Glucose+Ura	59.8	S2; 2B	S4
**S22**	RUC1665	*oriCX*	*dnaA1112*	Glucose+Ura	63.1	S2; 2B	S4

[1]) All cultures were grown in AB medium supplemented with (10 µg/ml) phenylalanine and the indicated carbon source (0.2 % for glucose and fructose, 0.5 % for glycerol). Uracil (20 µg/ml) was added if indicated.

Supplementary Table S3. Segmented replication fork speed (RFS) for *oriC* and *oriCX* strains.

	oriC	*oriC*	*oriC*	*oriC*	*oriC*	*oriCX*	*oriCX*	*oriCX*	*oriCX*	*oriCX*
C-source	glucose	glucose	fructose	glycerol	glycerol	glucose	glucose	fructose	glycerol	glycerol
L2 [1)]	0.78	0.67	0.64	0.57	0.60	0.72	0.65	0.74	0.78	0.83
X_L	0.79	0.68	0.58	0.60	0.61	0.53	0.43	0.40	0.42	0.40
X_R	0.86	0.72	0.67	0.78	0.73	0.54	0.43	0.41	0.44	0.42
C_L	0.81	0.83	0.66	0.62	0.65	0.51	0.42	0.38	0.43	0.41
C_R	0.81	0.87	0.67	0.63	0.66	0.52	0.43	0.39	0.43	0.42
R2	0.78	0.60	0.58	0.64	0.58	0.78	0.68	0.71	0.80	0.67
R3	0.82	0.78	0.64	0.67	0.74	0.88	1.03	0.79	1.01	1.05
Average RFS	0.81	0.73	0.63	0.64	0.65	0.62	0.53	0.50	0.54	0.53
O-P/O-D [2)]	1.01	1.24	1.07	0.97	1.01	0.70	0.64	0.55	0.53	0.55
oriX/*oriC* [3)]						0.99	1.00	0.99	0.98	1.00
Synchrony [4)]						1.01	1.00	1.00	0.97	1.00
Dt (min) [5)]	56.3	60.3	79.3	87.7	91.0	62.7	64.5	75.3	92.1	95.6
Sample [6)]	S1	S15	S5	S7	S11	S2	S17	S6	S8	S13

[1)] RFS (in kbp/s) for each segment as shown in Figure 1 in minimal medium with indicated carbon sources.

[2)] RFS of origin proximal segments (O-P: C_L and C_R for *oriC*; X_L, X_R, C_L, and C_R for *oriCX*) divided by RFS of origin distal segments (O-D: remaining segments).

[3)] Ratio of the read density at *oriX* divided by the read density at *oriC*.

[4)] RFS of X_L and C_R segments divided by RFS of segments between origins in the *oriCX* strain (X_R and C_L). Values near 1.00 indicate synchronous initiation at both origins.

[5)] Dt: doubling time.

[6)] Sample number from Supplementary Table S2.

Supplementary Table S4. Effect of uracil supplementation and the *dnaA1112* mutation on segmented RFS [1)].

Origin(s)	*oriC*	*oriC*	*oriCX*	*oriCX*	*oriC*	*oriC*	*oriCX*	*oriCX*
Uracil	-	+	-	+	-	+	-	+
dnaA allele	wt	wt	wt	wt	1112	1112	1112	1112
L2	0.67	0.80	0.65	0.74	0.90	1.02	1.05	0.91
X_L	0.68	0.70	0.43	0.49	1.10	1.06	1.01	1.22
X_R	0.72	0.93	0.43	0.51	1.25	1.29	1.28	1.80
C_L	0.83	0.88	0.42	0.49	1.54	1.83	1.28	1.69
C_R	0.87	0.90	0.43	0.50	1.68	2.13	1.11	1.32
R2	0.60	0.77	0.68	0.74	0.96	0.98	1.01	1.01
R3	0.78	0.80	1.03	1.09	1.31	1.22	2.25	1.19
Average RFS	0.73	0.82	0.53	0.61	1.22	1.27	1.17	1.16
% increase RFS (+/- uracil)		13%		13%		4%		0%
% increase RFS (*dnaA1112*/wt)					67%	55%	119%	92%
O-P/O-D	1.24	1.11	0.64	0.67	1.46	1.78	1.03	1.32
Synchrony			1.00	0.99			0.83	0.73
Dt (min)	60.3	54.6	64.5	59.8	67.0	60.4	65.90	63.1
% increase Dt (+/- uracil)		-9%		-7%		-10%		-4%
% increase Dt (dnaA1112/wt)					11%	11%	2%	6%
Sample	S15	S19	S17	S21	S16	S20	S18	S22

[1)] See Supplementary Table S3 for Table notes.

Supplementary Table S5. Effect of the *nrdR* deletion on segmented RFS [1)].

nrdR allele	wt	wt	wt	wt	*nrdR*::Kan	*nrdR*::Kan	*nrdR*::Kan	*nrdR*::Kan
Origin(s)	*oriC*	*oriC*	*oriCX*	*oriCX*	*oriC*	*oriC*	*oriCX*	*oriCX*
Carbon source	glucose	glycerol	glucose	glycerol	glucose	glycerol	glucose	glycerol
L2	0.78	0.57	0.72	0.78	0.99	0.83	0.78	0.83
X_L	0.79	0.60	0.53	0.42	1.05	0.83	0.88	0.70
X_R	0.86	0.78	0.54	0.44	1.19	1.27	0.96	0.78
C_L	0.81	0.62	0.51	0.43	1.22	0.96	0.87	0.76
C_R	0.81	0.63	0.52	0.43	1.28	1.02	0.86	0.74
R2	0.78	0.64	0.78	0.80	1.00	0.94	0.96	0.97
R3	0.82	0.67	0.88	1.01	1.10	0.97	0.93	0.98
Average RFS	0.81	0.64	0.62	0.54	1.11	0.96	0.89	0.80
% increase in RFS (*nrdR*::Kan/wt)					38%	50%	43%	47%
O-P/O-D	1.01	0.97	0.70	0.53	1.17	1.03	1.00	0.80
Synchrony			0.99	1.03			1.05	1.07
Dt (min)	56.3	87.7	62.7	92.1	59.2	94.4	65.5	98.7
	S1	S7	S2	S8	S3	S9	S4	S10

[1)] See Supplementary Table S3 for Table notes.

Supplementary Table S6. Effect of *dnaA* allele in glycerol medium [1)].

Origin(s)	*oriC*	*oriC*	*oriCX*	*oriCX*
dnaA allele	wt	1112	wt	1112
L2	0.60	0.84	0.83	0.69
X_L	0.61	0.84	0.40	0.83
X_R	0.73	1.49	0.42	1.12
C_L	0.65	1.03	0.41	1.11
C_R	0.66	1.12	0.42	0.91
R2	0.58	1.02	0.67	0.74
R3	0.74	1.03	1.05	1.07
Average RFS	0.65	1.03	0.53	0.86
% increase RFS (*dnaA1112*/wt)		57%		61%
O-P/O-D	1.01	1.03	0.55	1.22
Synchrony			1.00	0.78
Dt (min)	91	107.4	95.6	107.2
% increase Dt (dnaA1112/wt)		18%		12%
Sample	S11	S12	S13	S14

[1)] See Supplementary Table S3 for Table notes.

Supplementary Figures

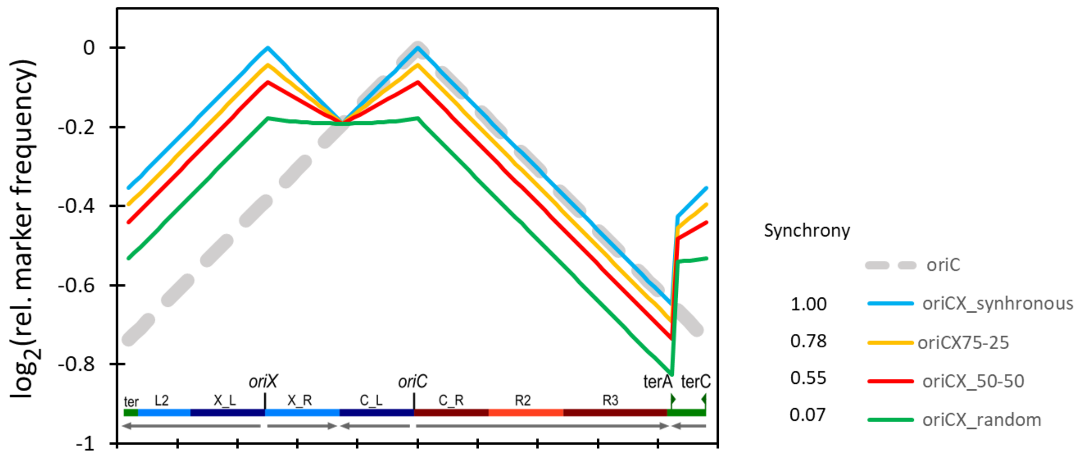

Supplementary Figure S1. Simulated replication profiles for *oriCX* with synchronous initiation at both origins in 100%, 75%, 50% or 0% of the cells in culture. Initiation in remaining cells is randomly at either origin. Simulated replication profile for *oriC* is shown for comparison. The synchrony index as defined by the apparent RFS of X_L and C_R segments divided by the apparent RFS of segments between origins in the *oriCX* strain (X_R and C_L) (Table 3) drops from 1.00 to 0.07 as shown.

Supplementary Figure 2: Additional replication profiles

A:

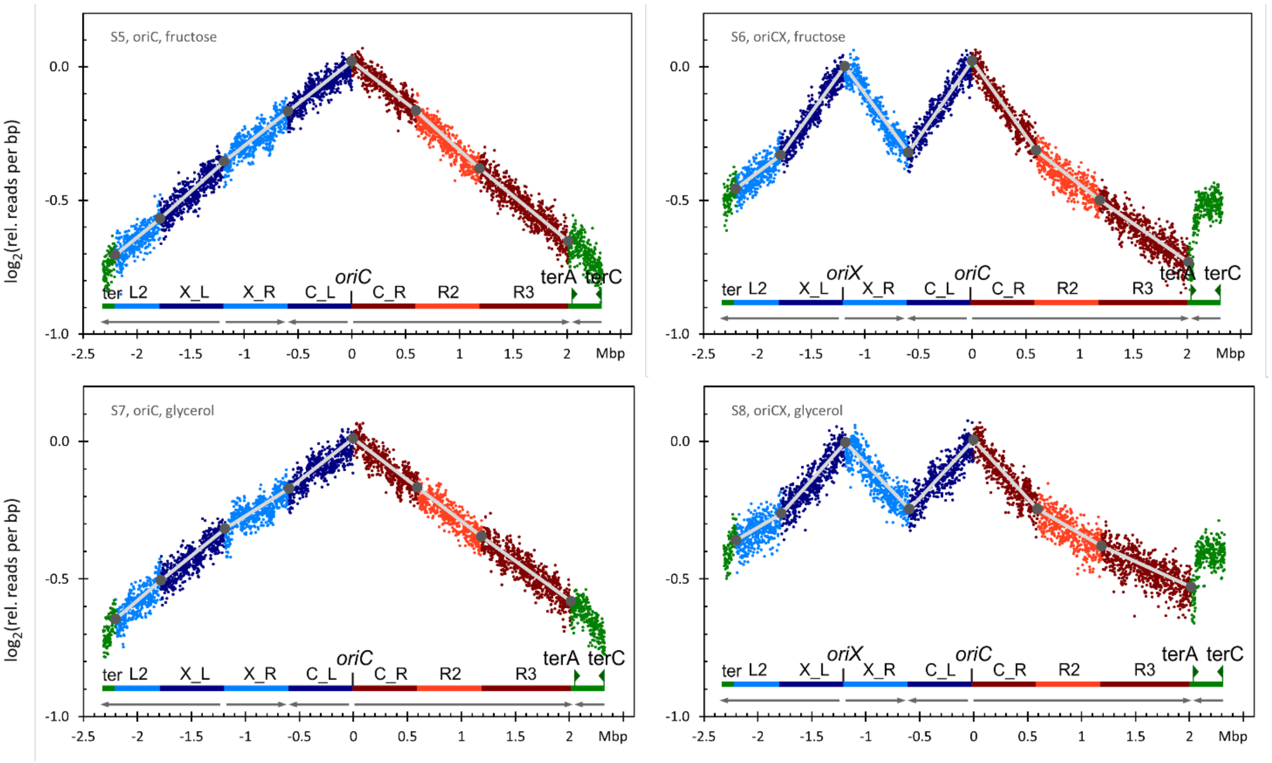

B:

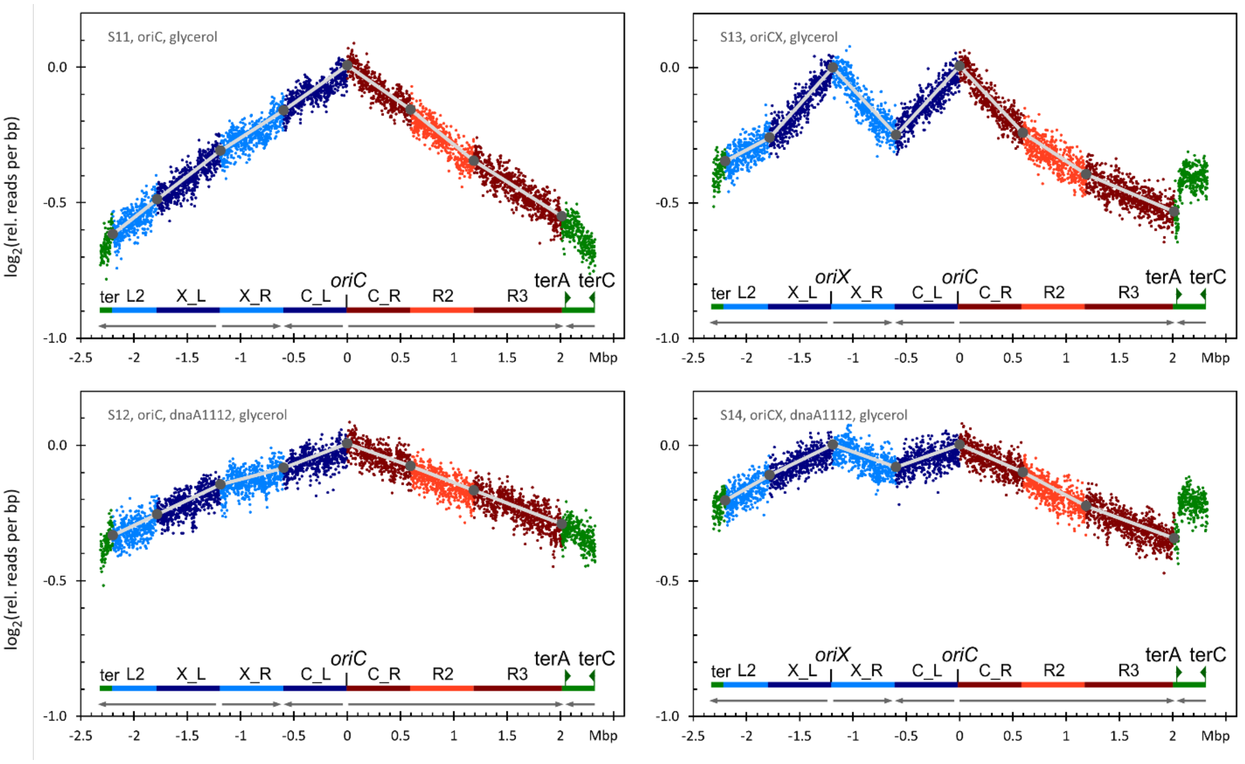

C:

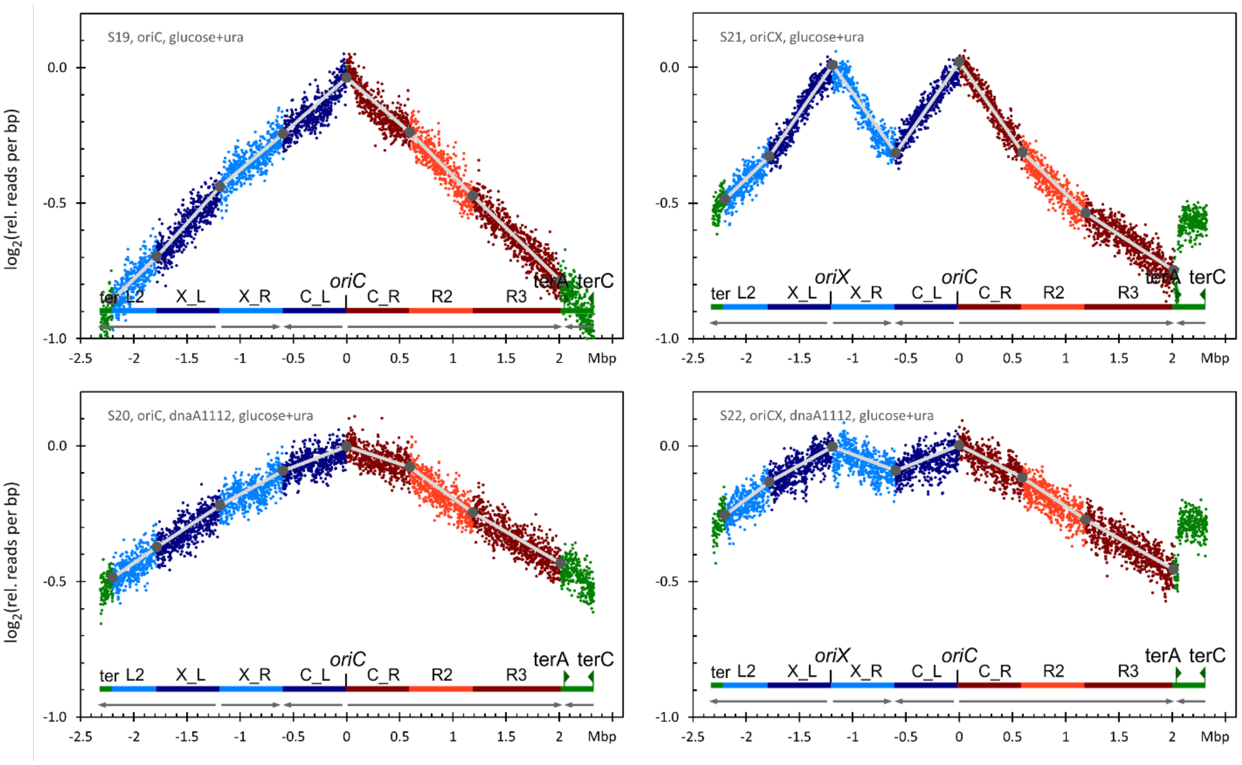

D:

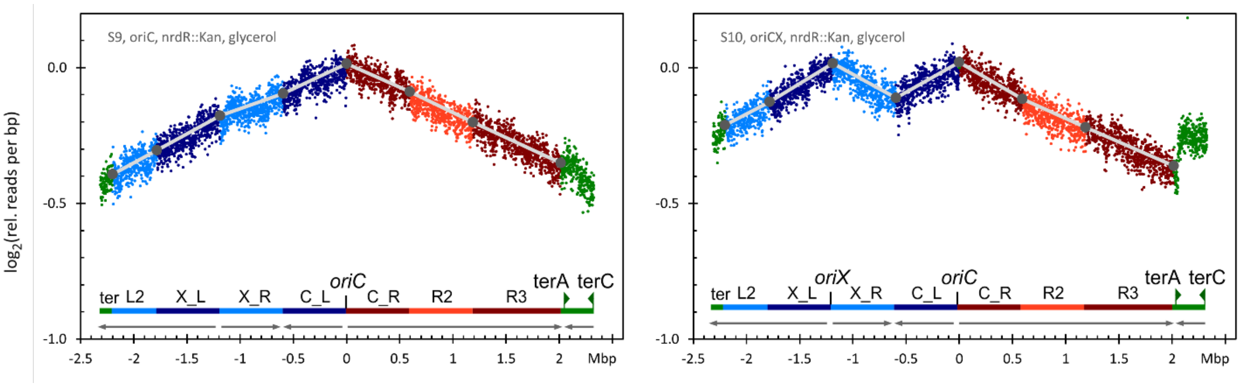

Supplementary Figure S2. (A) replication profiles of *oriC* and *oriCX* cultivated with fructose as carbon source to compare with **Figure 1C**. **(B)** replication profiles of *oriC* / *oriCX* combined with *dnaA*(wt) / *dnaA1112* cultivated with glycerol as carbon source to compare with **Figure 1C** (top panels) and with **Figure 2A. (C)** replication profiles of *oriC* / *oriCX* combined with *dnaA*(wt) / *dnaA1112* cultivated with glucose as carbon source and supplemented with uracil to compare with **Figure 1C** (top panels) and with **Figure 2A**. **(D)** replication profiles of *nrdR*::Kan combined with *oriC* / *oriCX* cultivated with glycerol as carbon source to compare with **Figure 3A**.

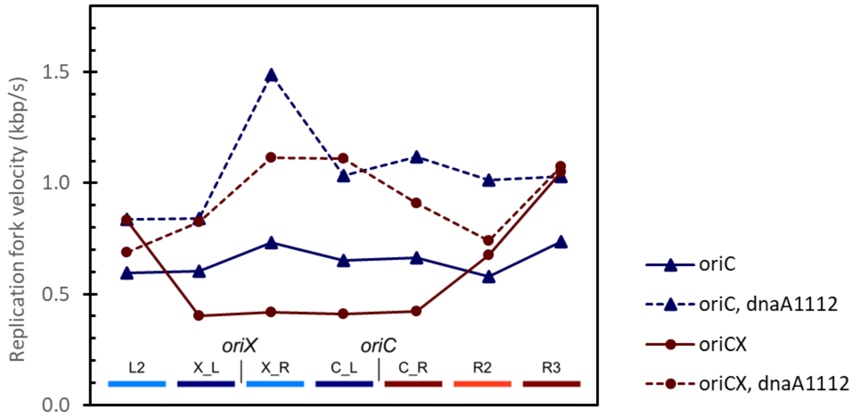

Supplementary Figure S3. Effect of *dnaA1112* mutation in glycerol medium. RFS plotted for each segment of *oriC* and *oriCX* combined with *dnaA*(wt) or width *dnaA1112* cultivated with glycerol as carbon source to supplement **Figure 2B**.

Supplementary references

Baba, T., Ara, T., Hasegawa, M., Takai, Y., Okumura, Y., Baba, M., Datsenko, K.A., Tomita, M., Wanner, B.L., and Mori, H. (2006). Construction of *Escherichia coli* K-12 in-frame, single-gene knockout mutants: the Keio collection. *Mol Syst Biol* 2**,** 2006 0008.

Dimude, J.U., Stein, M., Andrzejewska, E.E., Khalifa, M.S., Gajdosova, A., Retkute, R., Skovgaard, O., and Rudolph, C.J. (2018). Origins Left, Right, and Centre: Increasing the Number of Initiation Sites in the *Escherichia coli* Chromosome. *Genes (Basel)* 9.

Skovgaard, O., and Løbner-Olesen, A. (2005). Reduced initiation frequency from *oriC* restores viability of a temperature-sensitive *Escherichia coli* replisome mutant. *Microbiology* 151**,** 963-973.
